# Development of a Dihydrofolate Reductase Selection System for *Saccharomyces boulardii*

**DOI:** 10.3390/ijms26052073

**Published:** 2025-02-27

**Authors:** Hua Yu, Lydia Nyasae, Rachel Lee, Wenyan Lu, Edward So, Hanping Feng, Zhiyong Yang

**Affiliations:** Fzata Inc., 1450 S. Rolling Rd, Halethorpe, MD 21227, USA

**Keywords:** *Saccharomyces boulardii*, dihydrofolate reductase selection, methotrexate, sulfanilamide, tunable expression

## Abstract

*Saccharomyces boulardii*, the only commercially available probiotic yeast, has gained attention as a recombinant live biotherapeutic product (rLBP) empowered with the expression of heterologous therapeutic proteins for treating gastrointestinal diseases. However, the genetic modification of *S. boulardii* intended for clinical use is hindered by regulatory and technical challenges. In this study, we developed a dihydrofolate reductase (DHFR)-based selection system as an innovative alternative to traditional auxotrophic selection strategies for engineering *S. boulardii*. The DHFR selection system overcame inherent resistance of the yeast to methotrexate (MTX) by incorporating sulfanilamide, a dihydrofolate synthesis inhibitor, to enhance selection efficiency. The system demonstrated robust functionality, enabling the efficient screening of high-expression clones and tunable expression of therapeutic proteins, such as cytokines and antibodies, by modulating MTX concentrations. Furthermore, the yeast’s endogenous *DHFR* homolog, *DFR1*, was shown to be a viable selection marker, providing greater host compatibility while maintaining functionality compared to DHFR. This selection system avoids reliance on foreign antibiotic selection markers and the construction of auxotrophic strains, thus simplifying engineering and allowing for a tunable protein expression. These advancements establish the DHFR/DFR1 selection system as a robust and versatile platform for developing *S. boulardii*-based live biotherapeutics.

## 1. Introduction

*Saccharomyces boulardii* is a eukaryotic unicellular yeast, a subtype of *Saccharomyces cerevisiae* [1], widely recognized for its safe profile and probiotic benefits in gastrointestinal (GI) health. In the United States, *S. boulardii* is the only commercially available over-the-counter probiotic yeast for supporting intestinal functions, maintaining microbiota homeostasis, and promoting gut health [2]. In clinical studies, *S. boulardii* has shown efficacy in alleviating various GI disorders, including *Clostridioides difficile* infection (CDI), irritable bowel syndrome (IBS), and inflammatory bowel diseases (IBD), among others [3,4,5,6,7,8,9,10].

As a eukaryotic yeast, *S. boulardii* offers unique advantages over bacterial probiotics, particularly its insensitivity to most antibiotics, making it compatible with co-administration of antibiotics in clinical settings. Unlike *Saccharomyces cerevisiae*, *S. boulardii* thrives at 37 °C and demonstrates resistance to acidic environments, making it an ideal candidate for therapeutic applications in the human GI tract [11,12,13]. Recently, engineered *S. boulardii* as recombinant live biotherapeutic products (rLBPs) have been evaluated in preclinical studies and shown promising results against CDI [14], IBD [2,15], diabetes [16], obesity [17], and colon cancers [18].

Genetic modification of *S. boulardii* for therapeutic purposes needs to overcome several challenges, including regulatory ones. Exogenous antibiotic selection markers, which are widely used in scientific research, should be avoided according to FDA regulations. An alternative approach involves the use of auxotrophic markers, such as URA3, which relies on the deletion of genes crucial for growth in nutrient-limited conditions [19]. However, for obligate diploid strains like *S. boulardii*, this method requires inactivation of both gene alleles, making strain engineering a labor-intensive and complex process.

To address these challenges, we evaluated the dihydrofolate reductase (DHFR) selection marker system in *S. boulardii*. This system facilitates high and tunable gene expression through modulation of methotrexate (MTX) concentrations, which inhibits DHFR activity and induces cell death. The DHFR/MTX selection system has been widely adapted in mammalian expression systems, such as Chinese hamster ovary cells [20]. However, its application in *S. cerevisiae* is limited and remains unexplored in *S. boulardii* [21,22]. The inherent resistance of *S. boulardii* to MTX was mitigated by the inclusion of sulfanilamide, a compound that inhibits dihydrofolate synthesis, thereby enhancing the effectiveness of MTX-mediated selection.

In this study, we successfully introduced the DHFR selection marker system in *S. boulardii* for the first time and achieved high and tunable expressions of the genes of interest. Moreover, we evaluated the functions of both mammalian-derived *DHFR* gene and its yeast homolog, *DFR1* gene, as selection markers. This system allows rapid and efficient screening of strains with high and tunable expression of heterologous genes, paving the way for *S. boulardii* to emerge as a versatile platform for the next generation of rLBPs.

## 2. Results

### 2.1. DHFR Selection Is Feasible in S. boulardii

The DHFR selection system has not been previously applied to *S. boulardii*. To evaluate the feasibility of this system with *S. boulardii*, we conducted initial tests using a parental auxotrophic *S. boulardii* strain that bears a *URA3* deletion (Sb). The DHFR gene was integrated into a G418 selection plasmid (pCEV-G4-Km). *S. boulardii* was transformed with either the control plasmid (pCEV-G4-Km) or the DHFR plasmid (pCEV-G4-Km-DHFR). Transformants were successfully selected on YPD plates supplemented with 200 µg/mL G418 and were designated as Sb-G418 and Sb-G418-DHFR, respectively.

**Figure 1:**
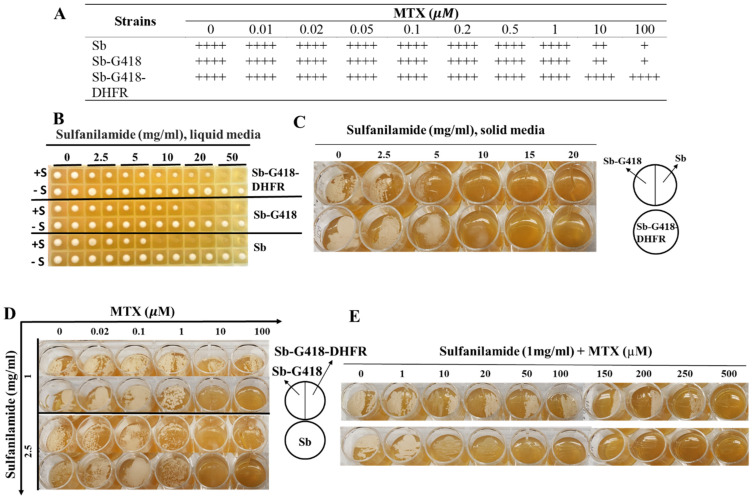
Dose optimization of MTX and sulfanilamide for DHFR selection. Sb-G418, Sb-G418-DHFR and control Sb strains were cultured in liquid YPD medium with or without 200 μg/mL of G418 for subsequent inoculations. (**A**) Cells were inoculated into a 96-deep well plate at an OD600 of 0.1 in liquid YPD supplemented with MTX ranging from 0–100 μM and cultured at 37 °C with shaking at 250 rpm for 24 h. Levels of growth are indicated by “++++” for robust growth, “++” for moderate growth, and “+” for low growth. (**B**) Cells were grown in YPD medium supplemented with sulfanilamide concentrations ranging from 0–50 mg/mL under the same condition as in (**A**). (**C**) Yeast strains were cultured on YPD agar media containing sulfanilamide at 0–20 mg/mL in a 24-well plate at 37 °C for 48 h. (**D**) Yeast cells were streaked onto YPD agar plate containing 0–100 μM MTX and with either 1 or 2.5 mg/mL sulfanilamide. Plates were incubated at 37 °C for 48 h. (**E**) Yeast cells were cultured on YPD agar plate containing 0–500 μM MTX with 1 mg/mL sulfanilamide and incubated at 37 °C for 48 h.

As expected, a differentiableresistance of Sb-G418-DHFR to MTX, compared to controls Sb and Sb-G418 lacking the exogenous DHFR, was observed in liquid culture. Since the endogenous *DFR1* gene of *S. boulardii*, the yeast equivalent of mouse *DHFR,* confers an inherent resistance to methotrexate (MTX), all strains exhibited robust growth at low MTX concentrations (Figure 1A). The growth rates of both Sb and Sb-G418 were reduced, but not completely inhibited, at higher MTX concentrations of 10 µM and 100 µM. In contrast, Sb-G418-DHFR maintained strong growth even at these higher MTX concentrations (Figure 1A). These results suggest that the enhanced resistance of Sb-G418-DHFR to MTX, relative to the controls, demonstrates its potential as a feasible selection marker for *S. boulardii*.

To enhance MTX effectiveness, sulfanilamide, which blocks the de novo synthesis of dihydrofolate [23], was included to refine the selection conditions. The above three strains were cultured in liquid media with varying concentrations of sulfanilamide to establish the optimal dose, followed by validation on solid media. In liquid culture, Sb grew slowly in the presence of increasing concentrations of sulfanilamide and ceased growing at sulfanilamide concentration of 10 mg/mL or higher (Figure 1B). Sb-G418, carrying the G418 selection gene encoding aminoglycoside 3′-phosphotransferase, demonstrated more resistance to sulfanilamide compared to Sb (Figure 1B). Sb-G418-DHFR exhibited the highest level of resistance to sulfanilamide with growth only inhibited at much higher sulfanilamide concentration of 50 mg/mL (Figure 1B). Similarly, Sb-G418-DHFR displayed higher resistance to sulfanilamide on solid media compared to the controls, but its growth was significantly inhibited at sulfanilamide concentrations exceeding 5 mg/mL (Figure 1C). Thus, sulfanilamide concentrations of 1 and 2.5 mg/mL were selected for further optimization of the selection dose in combination with varying doses of MTX.

In the presence of 2.5 mg/mL sulfanilamide, *S. boulardii* with or without DHFR formed colonies at MTX concentrations of 1 µM or lower but failed to grow at MTX concentrations at 10 µM or higher (Figure 1D). At these doses of sulfanilamide, the presence of exogenous DHFR was indistinguishable from controls. However, at a lower sulfanilamide concentration of 1 mg/mL, growth of both Sb and Sb-G418 was inhibited when MTX concentrations were 10 µM or higher, while Sb-G418-DHFR grew robustly under these conditions (Figure 1D; top panel). Based on these results, we tested 1 mg/mL sulfanilamide combined with escalated concentrations of MTX to define DHFR selection (Figure 1E). With sulfanilamide, MTX concentrations at 50 µM or higher completely inhibited the growth of yeast lacking exogenous DHFR expression. These findings indicate that in the presence of 1 mg/mL sulfanilamide, MTX at 50 µM or higher can be used for selecting DHFR-positive clones.

### 2.2. DHFR Selection Enables Screening for High-Expression Clones in S. boulardii

Building on these optimized conditions, we next evaluated the system’s ability to support stable and efficient expression of heterologous genes in *S. boulardii*. To achieve this, the cassette containing the GOI, together with DHFR selection marker, were integrated via site-specific homologous recombination into the *S. boulardii* genome. Several GOIs were used in this study, including a VHH gene targeting TNF-α fused with a human IgG1 Fc fragment, and a cytokine IL-22 gene fused with an HA tag. Previous studies have used inactive transposon delta sequences as site-specific genome integration loci to generate insertions of multiple copies of the GOIs [24]. We therefore used this strategy for genomic insertion of the GOIs in initial DHFR selection tests.

**Figure 2:**
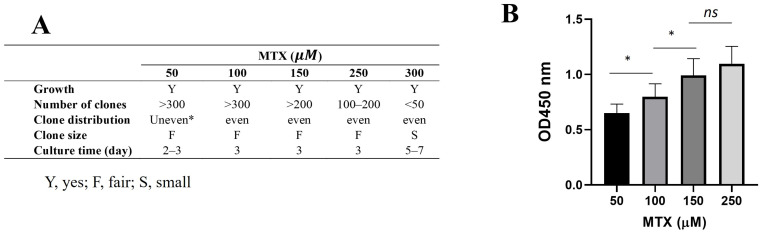
DHFR selection enables screening for high-expression clones in *S. boulardii*. (**A**) Yeast transformed with cassette containing murine IL-22 gene and DHFR selection marker was cultured on YPD agar plates containing the indicated MTX concentrations with 1 mg/mL sulfanilamide. Plates were incubated at 37 °C, and the colony parameters, including the number of colonies formed, colony distribution on the plate, colony size, and required culture time, were recorded. (**B**) Six colonies were randomly selected from the selection under the indicated concentrations of MTX and cultured in liquid YPD medium to assess murine IL-22 expression. The expression levels, as indicated by OD450 nm readings, in the culture supernatants were measured using ELISA. * denotes the *p*-value. A *p*-value < 0.05 indicates statistical significance. ns: not significant.

After transformation, equal number of yeast cells was inoculated on plates containing MTX ranging from 50 µM to 300 µM with 1 mg/mL of sulfanilamide. Higher concentrations of MTX resulted in a slower colony growth rate of transformants on the selection plates, reflected by both reduced colony density and smaller colony size (Figure 2A). At the highest MTX concentration tested (300 µM), fewer than 50 colonies formed, with a prolonged culture time of up to one week compared to 2–3 days at lower MTX concentrations. This observation suggests that excessively high MTX concentrations can impose cytotoxic effects on *S. boulardii*, compromising both growth and selection efficiency.

To investigate protein expression levels in the DHFR selection system, ELISA analysis of IL-22 expression from randomly selected clones revealed a positive association between higher MTX concentrations during selection and increased IL-22 expression levels (Figure 2B). Based on colony formation, growth rate, and protein expression levels, 250 µM of MTX with 1 mg/mL of sulfanilamide was identified as the optimal condition for the DHFR selection system for the rest of the studies. Under this condition, uniform colonies formed in comparable culture times to those observed at 50 µM MTX, but with higher protein expression levels. These results highlight the importance of carefully balancing MTX concentrations to maximize both selection efficiency and strain viability.

**Figure 3:**
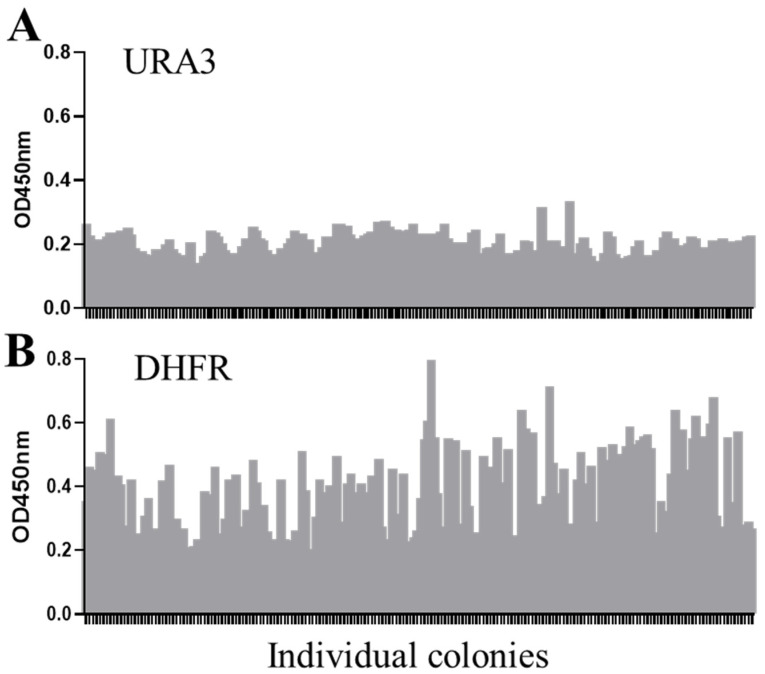
Comparison of protein expression levels from clones selected under DHFR or URA3 markers. The expression levels of the anti-TNF-α-Fc fusion proteins, indicated by OD450 readings, were determined by ELISA measurement of the cultured supernatants collected from individual colonies selected under URA3 (**A**) or DHFR (**B**) markers. Each bar represents a randomly picked individual colony and over 100 colonies for each condition were analyzed.

The traditional auxotrophic marker URA3 has been previously utilized as selection system for *S. boulardii* [25]. To directly compare DHFR selection with URA3 selection, yeast strains expressing anti-TNF-α-Fc were generated. The GOI cassettes were integrated into the yeast genome at the same locus and driven by a strong constitutive TEF1 promoter to directly compare the effects of DHFR selection versus URA3 selection in *S. boulardii*. Positive transformants were randomly picked from both selection groups, and the protein expression levels of individual clones were measured. DHFR selection (Figure 3B) resulted in a higher frequency of high-expression clones compared to URA3 selection (Figure 3A). This result demonstrates that the DHFR selection system was more likely to identify engineered strains with high GOI-expression relative to the URA3 auxotrophic marker system.

### 2.3. Endogenous Dihydrofolate Reductase DFR1 Can Be Used as a Selection Marker

**Figure 4:**
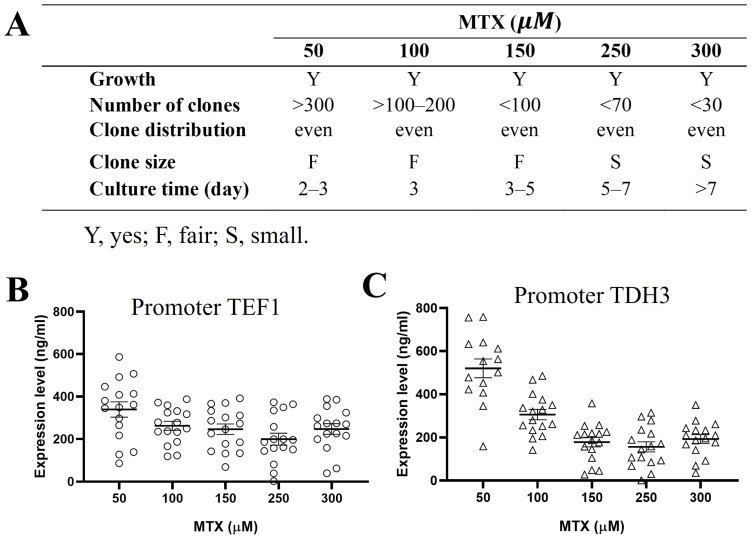
DFR1 as a selection marker. Yeast transformed with cassette containing an anti-TNF-α-Fc gene and DFR1 selection marker were plated on YPD agar containing MTX at 50 to 300 µM with 1 mg/mL sulfanilamide. Plates were incubated at 37 °C, and the colony parameters, including the number of colonies formed, colony distribution on the plate, colony size, and required culture time, were recorded (**A**). Individual colonies from (**A**) were picked and cultured in YPD overnight and the expression levels of anti-TNF-α-Fc, driven under either TEF1 promoter (**B**) or TDH3 promoter (**C**), were measured with quantitative ELISA. Purified anti-TNF-α-Fc expressed in *Pichia pastoris* (*Pichia* Expression Kit, Invitrogen, Catalog Number K1710-01) served as standard.

Next, we evaluated the potential of the yeast’s endogenous dihydrofolate reductase, DFR1, as an alternative selection marker in *S. boulardii*. The DFR1 system demonstrated similar functionality to DHFR under MTX selection, albeit with identifiable differences. DFR1 exhibited higher sensitivity to MTX, as evidenced by fewer positive transformants, smaller colony sizes, and longer culture times at higher MTX concentrations compared to DHFR selection (Figure 4A). We further measured the GOI expression levels from colonies selected under increasing MTX concentrations. The average expression levels of anti-TNF-α-Fc proteins decreased when the colonies were selected with MTX concentrations higher than 50 µM, regardless of whether the antibody was driven by the TEF1 promoter (Figure 4B) or TDH3 promoter (Figure 4C). Based on the results of the growth rate and the expression of the heterologous gene under the tested MTX concentrations, 50 µM MTX was determined as the effective dose for balancing transformant growth and protein expression in the DFR1system.

### 2.4. Protein Expression Level Is Correlated with GOI Copy Number in DHFR/DFR1 Selected Transformants

**Figure 5:**
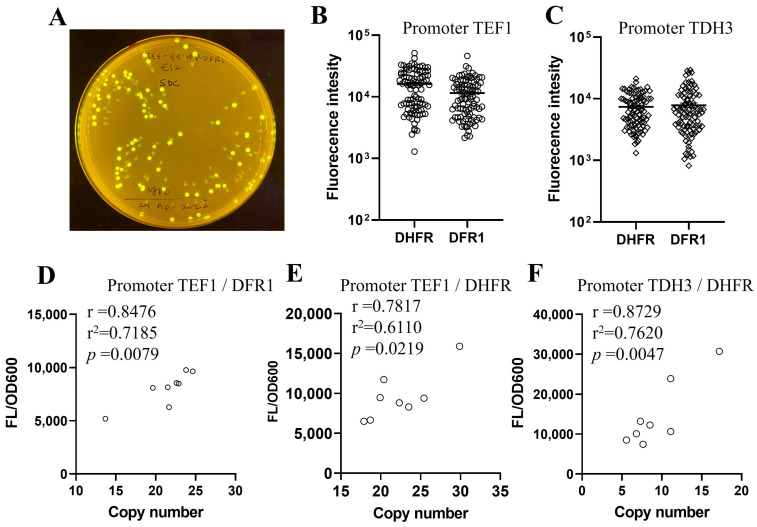
Correlation between protein expression levels and gene copy number in DHFR/DFR1 selection. (**A**) A representative image of EGFP-expressing transformants under DFR1 selection was captured under UV illumination of an agar plate containing 50 μM MTX and 1 mg/mL of sulfanilamide. (**B**,**C**) EGFP expression levels, indicated as fluorescent intensity, were comparable from random transformants selected under either DHFR or DFR1 markers. The EGFP genes were driven under either TEF1 (**B**) or TDH3 (**C**) promoters. (**D**–**F**) Pearson’s correlation was used to evaluate the association between EGFP expression levels and the gene copy numbers in transformants from the DFR1 selection (**D**) or the DHFR selection (**E**,**F**) under the TEF1 promoter (**D**,**E**) or the TDH3 promoter (**F**). r represents the correlation coefficient, which measures the strength and direction of a linear relationship between two variables and ranging from −1 to +1. Negative values would indicate an inverse correlation, while positive values indicate a direct correlation. r^2^, the coefficient of determination, represents the proportion of variance in the dependent variable explained by the independent variable(s), which ranges from 0 to 1. *p* denotes the *p*-value. A *p*-value < 0.05 indicates statistical significance.

The DHFR/DFR1 selection system was further validated using EGFP as a model protein. EGFP expression, easily detectable via fluorescence microscopy and quantifiable through fluorescence measurements, provided a reliable means of evaluating the system. An EGFP reporter system was constructed and integrated into the *S. boulardii* genome following the same methodology used for other expression cassettes. As shown in Figure 5A, all colonies demonstrated detectable EGFP expression under UV light, confirming the consistency of the selection system. EGFP expression levels among clones selected with either DHFR or DFR1 were comparable under either P_TEF1_ (Figure 5B) or P_TDH3_ (Figure 5C) promoter.

To explore the relations between EGFP expression levels and the gene copy numbers integrated into the yeast genome, qPCR analysis of eight randomly selected colonies from DFR1 or DHFR transformants was performed. A significant positive correlation was observed between EGFP fluorescence intensity and the copy number of the inserted gene cassette (Figure 5D–F). These results validate that high copy numbers of integrated GOI correlated with elevated protein expression levels. Therefore, the DHFR/DFR1 selection system facilitates multi-copy insertions of the gene expression cassette with tunable expression levels in *S. boulardii*.

### 2.5. Gene Amplification Can Be Achieved by MTX in DHFR Selection

MTX pressure is widely employed in mammalian systems as a high-throughput screening strategy to select high-yield, stable-expression clones. This study evaluated whether a similar strategy could enhance GOI copy number and expression levels in *S. boulardii*. Since *S. boulardii* is sensitive to a broader range of MTX concentrations in DHFR selection compared to the DFR1 selection, the impact of MTX pressure was tested using the DHFR selection system.

**Figure 6:**
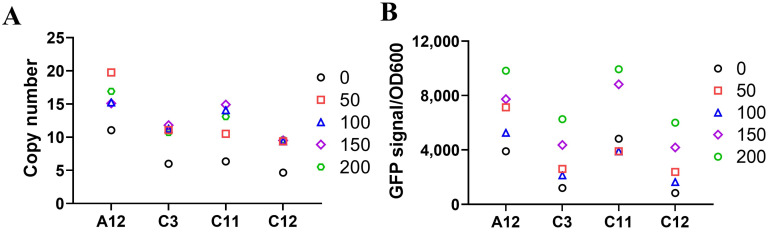
Gene amplification and protein expression under MTX selection pressure. Four clones with low to medium copy numbers (≤15 copies) of the EGFP gene were cultured in liquid YPD containing 1 mg/mL sulfanilamide and different concentrations of MTX from 0 to 200 µM for two weeks with daily passage and medium change. The same medium was used for passage and initial seeding density of passage is OD600 of 0.5. After two-week culture, the EGFP gene copy number (**A**) and corresponding EGFP expression levels (**B**) were quantified for the pooled cells under each MTX-concentration condition.

Clones with low to medium copy numbers of the EGFP gene were selected to evaluate the effect of prolonged MTX pressure. The selected clones were cultured in liquid media containing 1 mg/mL sulfanilamide and increasing concentrations of MTX to observe the changes in gene copy numbers and protein expression levels. Up to 2 weeks of exposure to increasing MTX concentrations resulted in amplified gene copy numbers (Figure 6A) and a corresponding increase in EGFP expression levels (Figure 6B) across individual yeast clones, with a varying degree of response among different clones. Therefore, gene amplification was achieved by continued MTX pressure on those clones with low to medium GOI copy numbers in the yeast genome.

## 3. Discussion

This study presents the first successful application of the DHFR selection system in *S. boulardii*, marking an important advancement in the development of genetically engineered probiotic yeast for biotherapeutic purposes. The DHFR system is an effective selection marker for *S. boulardii*, facilitating high-throughput screening of high-expression clones. Through optimization of MTX concentration in combination with sulfanilamide, we effectively established a non-antibiotic and non-auxotrophic selection system specifically designed for therapeutic applications.

Compared to traditional auxotrophic selection markers like URA3, the DHFR system offers unique advantages. The system can be applied directly to wild type *S. boulardii* avoiding the labor-intensive process of constructing auxotrophic strains. Our results demonstrate that DHFR/DFR1 selection outperformed auxotrophic selection by yielding a higher frequency of clones with enhanced protein expression. This strategy simplifies the genetic engineering of *S. boulardii* while adhering to regulatory guidelines that discourage the use of foreign genetic elements in therapeutic microorganisms with strengthened protein expression.

In this study, we found that clones with different GOI gene copy numbers and expression levels could be obtained by modulating MTX concentrations in the selection media. Together with the strong correlation between gene copy number and protein expression as determined in the EGFP reporter system, this study highlights the utility of DHFR selection for tunable expression of exogenous proteins. Moreover, this fine tunability, achieved by modulating MTX concentrations, allows for isolation of yeast clones with variable expressions levels, a feature particularly valuable in the production of therapeutic proteins where accurate control of dosage is critical for efficacy and safety.

The mouse DHFR-based and yeast DFR1-based selection system share functional similarities, but key differences in their responses to MTX concentrations were observed. While higher MTX concentrations in the DHFR system enhanced gene amplification and protein expression, the same MTX concentrations in the DFR1 system led to a reduced protein expression possibly due to heightened sensitivity to MTX. Notably, *S. boulardii* exhibited inherent resistance to MTX, conferred by endogenously produced DFR1, necessitating the inclusion of sulfanilamide to enhance MTX selection efficiency. Future studies may evaluate MTX combining different amounts of sulfanilamide or alternative compounds to sulfanilamide for DFR1 selection. Such investigations will be crucial for refining the DFR1 selection strategy and advancing yeast-based selection systems for diverse biotechnological applications.

The development of this selection system positions *S. boulardii* as a suitable vehicle for delivering therapeutic proteins. Engineered strains expressing proteins, such as cytokines or antibodies, could target immune functions, potentially modulating GI inflammatory diseases and related disorders. With growing interest in live vectors for drug delivery, the DHFR-based selection system provides a scalable and efficient strategy for the production of *S. boulardii*-based rLBPs. This system is particularly well-suited for applications in GI diseases, where targeted delivery is critical for achieving better therapeutic efficacy.

## 4. Materials and Methods

### 4.1. Strains, Media, and Culture Conditions

*S. boulardii* (ATCC MYA-796 *ΔURA3*) was used as the parental strain for genetic modifications. *Escherichia coli* strain DH5α was used for plasmid production. *E. coli* transformed with recombinant plasmids were selected in LB medium (MBPE-4040, Growcells.com, Irvine, CA, USA) supplemented with Ampicillin (100 µg/mL) (61-238-RH, Corning, Glendale, AZ, USA).

Yeast was cultured using YPD (2% Glucose [GB0219, BioBasic, Markham, ON, Canada], 20 g/L peptone [97064-186, VWR, Radnor, PA, USA], and 10 g/L yeast extract [BT217100, VWR, Radnor, PA, USA]) liquid medium or agar plates. For antibiotic selection, G418 disulfate (Caisson Labs, Smithfield, UT, USA) at 200 µg/mL was used. To optimize DHFR/DFR1 selection conditions, a single positive colony from the G418 selection plate was cultured in liquid YPD containing G418 (200 µg/mL), then inoculated into either liquid or agar YPD supplemented with various concentrations of sulfanilamide (Alfa Aesar, Ward Hill, MA, USA) or methotrexate (MTX; Sigma-Aldrich, St. Louis, MO, USA), or their combination, and cultured at 37 °C for 48 h or at the indicated time.

Yeast was transformed with the transgene cassettes containing the gene of interest (GOI) and a DHFR/DFR1 selection marker, after which positive transformants were isolated on YPD agar plates supplemented with 1 mg/mL sulfanilamide and a minimum of 50 µM MTX. To identify high expression transformants, 250 µM or 50 µM MTX was used for DHFR or DFR1 selection, respectively, in combination with 1 mg/mL sulfanilamide. Plates were incubated at 37 °C for 2–3 days.

For strain expansion, single colonies were seeded into synthetic minimal (SM) medium (2% glucose, 6.8 g/L yeast nitrogen base without amino acids but with ammonium sulfate (Y0626, Sigma-Aldrich, St. Louis, MO, USA), 2 g/L Yeast Synthetic Drop-out Medium Supplements without Uracil (Y1501, Sigma-Aldrich, St. Louis, MO, USA) supplemented with 76 mg/L uracil (U0750, Sigma-Aldrich, St. Louis, MO, USA)) to support the growth of URA3 auxotrophic strains.

For target gene expression, selected clones were cultured in liquid YPD with optimized selection conditions at 37 °C for 24 h.

### 4.2. Plasmid Construction

The plasmid pCEV-G4-Km (Plasmid #46819, Addgene, Watertown, MA, USA) was used as the backbone to generate dual gene expression cassette plasmids with antibiotic selection markers. The plasmids pCEV-G4-DHFR/DFR1 were constructed by insertion of DHFR/DFR1 cassette into the plasmid backbone using specific restriction enzyme sites. The DHFR cassette was created by cloning a murine *DHFR* gene (UniProt# P00375) synthesized by Genscript (Piscataway, NJ, USA) under the URA3 promoter. The DFR1 cassette was constructed by amplifying the full-length *DFR1* gene from wild-type MYA-796 using Phusion^®^ High-Fidelity PCR Master Mix with HF Buffer (M0531L, New England Biolabs, Ipswich, MA, USA), following the manufacturer’s protocol.

Using these plasmids containing the selection cassette, we designed transgene cassettes encoding various GOIs for evaluation. These GOIs included a single-domain antibody (VHH) fused with human Fc fragments specific TNF-α (anti-TNF-α-Fc), an HA-tagged murine IL-22, and an enhanced green fluorescent protein (EGFP). A previously used AT signaling peptide [14] was used to drive the secretion of anti-TNF-α-Fc and IL-22, whereas no signaling peptide was used for EGFP for intracellular expression. The GOIs were cloned under either TEF1 or TDH3 promoter of *S. boulardii* and incorporated into pCEV-G4-DHFR/DFR1 to establish pCEV-GOI-DHFR/DFR1 plasmids using Gibson assembly [26]. All GOI’s were synthesized by Genscript (Piscataway, NJ, USA).

### 4.3. Yeast Transformation/Electroporation

Yeast cells were transformed using a high-efficiency lithium acetate protocol with 500 ng of plasmid per reaction as described previously [14]. For chromosomal integration, DNA fragments containing GOI and selection markers were PCR-amplified using the plasmids as templates and electroporated into the yeast for site-specific insertion into the yeast genome facilitated by homologous recombination [27].

### 4.4. ELISA

The expression levels of the GOIs from yeast clones selected using either DHFR/DFR1 or URA3 selection were determined by ELISA. For IL-22, the target protein in the yeast supernatants was captured by a rabbit anti-mouse IL-22 antibody (PA5-47782, Invitrogen, Carlsbad, CA, USA) and detected using an HRP-conjugated anti-HA tag antibody (PA129751, Thermo Fisher Scientific, Waltham, MA, USA). To detect anti-TNF-α-Fc, the fusion proteins were captured with recombinant TNF-α (TNA-H4211, ACROBiosystems, Newark, DE, USA) and detected with an HRP-conjugated goat anti-human IgG gamma antibody (H10007, Thermo Fisher Scientific, Waltham, USA).

### 4.5. EGFP Expression Studies

To assess EGFP expression, single colonies isolated from the selection plate were cultivated in 500 µL of YPD medium in a 96-deep-well plate at 37 °C for 24 h. After culture, cells were pelleted by centrifugation and washed twice with distilled water to remove residual media components. The cell pellet was then resuspended in distilled water and 100 µL of cell suspensions were transferred into a white plate for fluorescence analysis. Mean fluorescence intensity (MFI) was measured using an ELISA plate reader (Synergy H1 Microplate Reader, BioTek, Winooski, VT, USA) with an excitation wavelength of 488 nm and an emission wavelength of 528 nm. Mean absorbance at 600 nm (OD600) was recorded to determine cell density. EGFP expression was normalized by calculating the ratio of fluorescence intensity to OD600 (EGFP signal/OD600).

### 4.6. EGFP Copy Number

Real-time quantitative PCR (qPCR) was employed to determine the copy number of *EGFP* genes inserted in the genome of *S. boulardii*. Primers specific to the promoter sequences of target gene P_TEF1_ or P_TDH_ and the reference gene *TAF10* were designed using Primer3Web (https://primer3.ut.ee/, accessed on 12 October 2023). Genomic DNA (gDNA) was extracted from EGFP transformed yeast (test) or parental yeast (calibrator) using Quick-DNA Fungal/Bacterial Microprep Kit (Zymo Research, Irvine, CA, USA) following the manufacturer’s protocol. qPCR reactions were performed using PowerUp™ SYBR™ Green Master Mix (A25776, Fisher Scientific, USA) and 1 ng of gDNA as template. Reactions were performed on a QuantStudio 3 Real-Time PCR system (Applied Biosystems, Foster City, CA, USA) under the following thermocycling conditions: an initial denaturation at 95 °C for 15 s, followed by 40 cycles of denaturation at 95 °C for 15 s and annealing/extension at 60 °C for 60 s. The *EGFP* gene copy numbers were determined as the total copy numbers of its promoter, either P_TEF1_ or P_TDH_, minus the two endogenous copies. Data analysis was performed using Design and Analysis Software v2.7 (Software Information, Thermo Fisher Scientific, Waltham, MA, USA), and calculation of relative gene copy numbers was based on the ∆∆Ct method.

### 4.7. Statistics

A Pearson’s correlation analysis was used to examine the association between expression levels and GOI copy numbers. Statistical analyses were performed using GraphPad Prism 8 software (GraphPad Software, San Diego, CA, USA).

## 5. Conclusions

This study demonstrates the successful application of the DHFR/DFR1 selection system in *S. boulardii* for the first time and highlights its potential in producing bioengineered probiotic yeast as live biotherapeutic products. By optimizing MTX selection pressure, a tunable high-level expression of therapeutic proteins was achieved, which may facilitate the development of rLBPs.

## Data Availability

Data that support the findings of this study are available from the corresponding author upon reasonable request.
